# The Roche Total Mycophenolic Acid® assay: An application protocol for the ABX Pentra 400 analyzer and comparison with LC–MS in children with idiopathic nephrotic syndrome

**DOI:** 10.1016/j.plabm.2016.12.002

**Published:** 2017-01-04

**Authors:** François Parant, Bruno Ranchin, Marie-Claude Gagnieu

**Affiliations:** a Hospices Civils de Lyon, GHS – Centre de Biologie Sud, UM Pharmacologie – Toxicologie, Pierre Bénite F-69495, France; b Hospices Civils de Lyon, Hôpital Femme Mère Enfant, Service de Néphrologie et Rhumatologie Pédiatriques, Bron F-69677, France

**Keywords:** Enzyme assay, Inosine monophosphate dehydrogenase, Mycophenolic acid, Nephrotic syndrome

## Abstract

**Background:**

For TDM of mycophenolate acid (MPA), the Roche Total Mycophenolic Acid® assay based on the inhibition of recombinant inosine monophosphate dehydrogenase (IMPDH) has been shown to be a simple and reliable alternative to chromatographic methods. We have adapted this assay on the ABX Pentra 400 analyzer (HORIBA).

**Objective:**

To investigate the analytical performances of the Roche Total Mycophenolic Acid® assay on the ABX Pentra 400 and to compare it to an LC-MS method using samples from children with nephrotic syndrome treated with mycophenolate mofetil (MMF).

**Material and methods:**

Configuration of the open-channel on the ABX Pentra 400 was based on the Roche MPA assay package insert. Precision was determined as described in the CLSI protocol EP5-A2. Comparison with the LC-MS method was performed using 356 plasma samples from 42 children with nephrotic syndrome (8 h pharmacokinetic profiles).

**Results:**

The enzymatic assay demonstrated high precision. The %CV for Within Run Imprecision ranged from 5.5% at 1.2 mg/L to 1.5% at 14.1 mg/L and Total Imprecision ranged from 9.3% to 2.5%. The method comparison with plasma samples from children yielded overall a good correlation and a good agreement between both methods. The Passing Bablok regression analysis showed the following results: [Roche MPA assay]=1.058 [MPA LC-MS] −0.06; rho=0.996.

**Conclusion:**

The Roche Total Mycophenolic Acid® assay is adaptable to the ABX Pentra 400 analyzer, and demonstrates accurate and precise measurement of MPA in plasma obtained from children with nephrotic syndrome.

## Highlights

• Adaptation of the Roche Total Mycophenolic Acid® assay to the Pentra 400 analyzer.
• Comparison with LC-MS in children with idiopathic nephrotic syndrome.
• Therapeutic drug monitoring of mycophenolate mofetil.

## 1 Introduction

Since its regulatory approval in 1995, mycophenolate mofetil (MMF) has largely replaced azathioprine as the anti-metabolite immunosuppressant of choice in solid organ transplantation [1]. Over recent years, MMF has also increasingly been used off-label in adults for a number of indications, including autoimmune conditions [2], and to prevent relapses of idiopathic nephrotic syndrome in pediatric patients [3], [4], [5].

After oral administration, MMF is extensively hydrolyzed to mycophenolic acid (MPA) by esterases in the stomach, small intestine, blood, liver, and tissues. MPA displays complex pharmacokinetics with substantial between-subject variability. MPA is metabolized by uridine diphosphate glucuronyl transferases to mycophenolic acid glucuronide (MPAG) and to a minor metabolite, the acyl glucuronide (AcMPAG). MPA undergoes enterohepatic circulation as a result of biliary excretion of MPAG and deconjugation to MPA in the gastrointestinal tract. Elimination of MPAG and AcMPAG occurs via renal excretion [6].

Various studies suggest that a link may exist between plasma MPA concentrations and the risk of allograft rejection. Strategies to individualise dosage and optimise treatment with MMF are therefore important. A consensus report published in 2010 made some preliminary recommendations about Therapeutic Drug Monitoring (TDM) of MPA in solid organ transplantation [7]. Over recent years, there has also been increasing interest in TDM of MPA in non-transplant indications such as lupus nephritis [2], [8] and pediatric nephrotic syndrome [9], [10], [11], [12], [13]. In children with frequently relapsing steroid-sensitive nephrotic syndrome, achieving an area under the MPA concentration-time curve in the first 12 h (MPA-AUC0–12 h)>50 mg h/L not only had significantly fewer relapses than patients with an MPA-AUC0–12 h<50 mg h/L (1.4 versus 0.27 per year), but also a similar number of relapses and a similar relapse-free observation time to CsA-treated patients [9]. In this study, MPA was measured by a Cloned Enzyme Donor Immunoassay (CEDIA) which cross-reacted with the acyl glucuronide (AcMPAG) and led to over-estimation of MPA concentrations by 36% on average [14].

Chromatographic methods, especially when combined with tandem mass spectrometry, do not suffer from this disadvantage and have become the standard procedure to quantify MPA [15], [16], [17]. But they are not available everywhere, and often require time-consuming sample preparation. Recently, an automated enzymatic assay based on the inhibition of recombinant inosine 5′-monophosphate dehydrogenase (IMPDH) was designed to measure plasma MPA concentrations. Comparisons of this technique with chromatographic methods showed an excellent correlation in transplant recipients [18], [19]. However, this enzymatic assay has only been evaluated on Roche COBAS C and COBAS INTEGRA analyzers.

Here, we report the adaptation of the Roche Total Mycophenolic Acid® assay to the ABX Pentra 400 analyzer and its comparison with liquid chromatography-mass spectrometry (LC-MS) in children with idiopathic nephrotic syndrome treated with MMF.

## 2 Materials and methods

### 2.1 Enzymatic assay

The Roche Total Mycophenolic Acid® (Roche Diagnostiocs, Rotkreuz, Switzerland) assay is based on specific binding of MPA to its enzyme/receptor target, IMPDH Type II (IMPDH II). IMPDH II catalyzes the oxidation of inosine monophosphate (IMP) to xanthosine monophosphate (XMP); the reaction is coupled with the reduction of NAD to NADH. The presence of MPA in the sample inhibits the enzymatic reaction, resulting in decreased rate of NADH production, which can be determined by monitoring absorbance at 340 nm. The reagent format consists of two liquid ready to use reagents in which R1 (enzymatic reagent) contains recombinant IMPDH II in buffer (15.7 U/L), and IMP, and R2 (substrate reagent) contains NAD in buffer (10 mmol/L).

### 2.2 Adaptation to the ABX Pentra 400 analyzer

The ABX Pentra 400 analyzer (Horiba Ltd, Kyoto, Japan) is a compact bench top clinical biochemistry analyzer [20]. The configuration of the open channel was based on the Roche Total Mycophenolic Acid® assay package insert. Briefly, the analyzer added 3 µL of sample to 180 µL of R1 (cycle 1; a cycle every 12 s). After mixing and incubation for 5 min, 19 µL of R2 was added and mixed (cycle 26). At this point, bichromatic measurements (340/405 nm) were performed. The slope of the reaction rate is determined between cycles 49 and 62. The measuring temperature (37 °C) was controlled by an air bath. The assay was calibrated with 6-point calibration at 0, 1, 3, 5, 10 and 15 mg/L (Roche Total MPA Calibrators; Roche Diagnostics) using a logit/log4 calculation mode. All samples above 15 mg/L were manually diluted (1:3) with the diluent (equivalent to the 0 mg/L calibrator) from the Roche Total MPA Calibrators.

The assay was bidirectionally interfaced to the Laboratory Information System (LIS) using barcoded tubes. The reagents were stored in the integrated refrigerated reagent compartment (2–10 °C).

### 2.3 Validation procedures

#### 2.3.1 Functional sensitivity

The functional sensitivity corresponds to the minimum concentration giving a total imprecision coefficient of variation (CV) of 20% and is used to establish the Low Limit of Quantification (LLOQ) for the MPA enzymatic assay [21]. 5 samples with low MPA concentrations (0.18 mg/L to 1.25 mg/L) were tested daily over 20 days (20 determinations in all) and total imprecision %CVs were determined. The functional sensitivity was calculated by plotting the imprecision profile of the method using Variance Function Program, version 2016 (Bill Sadler, Christchurch, New Zealand).

#### 2.3.2 Precision

The precision study was determined using the guidelines of the Clinical and Laboratory Standards Institute (CLSI, formerly NCCLS), Protocol EP5-A2 "Evaluation of Precision Performance of Clinical Chemistry Devices, Approved Guideline – Second Edition" [22]. A first imprecision study according to CLSI protocol EP5A2 was performed by using Siemens EMIT® 2000 tri-level MPA Controls (Siemens Healthcare Diagnostis, Erlangen, Germany) (aqueous/bovine serum albumin matrix) (February to March 2009) and a second by using Roche Total tri-levels MPA Controls (Roche Diagnostics; human serum matrix) (April to May 2010).

Within-run and total imprecisions were calculated by Analyze-It Software version 2.22 for Microsoft Excel (Analyse‐it Software Ltd, Leeds, UK) and compared against published data.

#### 2.3.3 Accuracy

The method accuracy was assessed using External Quality Assessment (EQA) “Mycophenolate International Proficiency Testing (IPT) Scheme” from Analytical Services International, London, UK (www.bioanalytics.co.uk).

#### 2.3.4 Quantitative statement of the uncertainty in measurement (UM)

The UM was estimated using the long-term evaluation of the UM (LTUM) [23]. The LTUM method is based on linear regression between data obtained by participants in EQA schemes and target values. The LTUM approach is a simple and convenient method that gives UM estimates that are reliable and comparable to the French accreditation body (COFRAC) recommended approach (SH GTA 14 IQC/EQA method) [24].

#### 2.3.5 Carryover effect

Carryover was assessed by running a high MPA concentration sample three times (H1-H2-H3) followed by a low MPA concentration sample three times (L1-L2-L3). Carryover % is calculated by: (L1-L3)/(H3-L3)×100.

### 2.4 Comparison with liquid chromatography-mass spectrometry (LC-MS) method

Values obtained by enzymatic assay were plotted against those obtained by a Liquid Chromatography-Diode Array Detection/Electrospray Ionization-Mass Spectrometry performed using an Agilent 1100 Series G1956B LC/MSD instrument.

#### 2.4.1 Sample sources

Comparison with the chromatographic method was performed using plasma samples from children with nephrotic syndrome (full pharmacokinetic profiles collected at 0, 20 min, 40 min, 1 h, 2 h, 3 h, 4 h, 6 h and 8 h after a morning dose of mycophenolate mofetil). The samples were derived from children included in a MMF pharmacokinetic study (from July 2008 to April 2009). The aim of this study was to design Bayesian estimators for forecasting pharmacokinetics and dose adjustment using a limited number of blood samples [11].

#### 2.4.2 Preanalytical conditions

Whole blood EDTA samples reached the laboratory no later than 10–12 h after sampling. Samples were then centrifuged, aliquoted and stored at 2–8 °C (aliquot for enzymatic method) or −20 °C (aliquot for chromatographic method). Plasma were analyzed by enzymatic method the same day or the next morning at the latest, and analyzed by chromatographic method within 7 days.

#### 2.4.3 Sample preparation for LC-MS

One hundred µL of sample (calibrator, control, and patient plasma specimen) dispensed into a 1.5-mL polypropylene microcentrifuge tube were mixed with 30 µL of internal standard solution (10 mg/L indomethacin in methanol/water [10% v/v]), and 200 µL of acetonitrile. The mixture was acidified with 10 µL of 99% pure formic acid was vortexed twice for 15 s and centrifuged at 17,900 rcf for 5 min. The resulting supernatant was transferred into a new microcentrifuge tube and evaporated to dryness under nitrogen. The residue was dissolved in 100 µL of the mobile phase and centrifuged again. Seventy µL of the resulting solution was transferred to an HPLC vial and 5 µL was injected into the LC-ESI-MS system.

#### 2.4.4 Chromatography and mass spectrometry

Chromatographic separation was achieved by isocratic elution with a mixture of acetonitrile (45%) and ammonium formate buffer (4 mM, pH 3.75) pumped at a flow rate of 0.6 mL/min on a SymetryShield™ RP8 column (3.5 µm 2.1×150 mm; Waters Corporation, Milford, MA, USA) thermostatted to 50 °C. Electrospray ionization conditions were set as follows: the fragmentor voltage was 100 V, the nitrogen gas flow was maintained at 13 L/min, the nebulizer pressure was set up at 60 psig, the positive capillary voltage was 4.5 kV and the drying gas temperature was 350 °C. Quantification was performed in the positive selected-ion monitoring (SIM) mode using target ions at [M+H]^+^ *m*/*z* 321 for MPA and [M+H]^+^ *m*/*z* 358 for the internal standard. Their retention times were 3.9 min and 8.5 min, respectively. The calibration curve consisted of three calibrators plus a zero calibrator of drug-free serum (Recipe®, Munich, Germany). It should be noted that this procedure does not correspond to current criteria for calibration (six to eight non-zero samples covering the expected range, including LLOQ) and may limit the quality of the regression analysis.

#### 2.4.5 Analytical performance characteristics

The low limit of quantification (signal-to-noise ratio of 10) was 0.07 mg/L and the calibration curve was linear up to 80 mg/L. The total imprecision, calculated by measuring the CVs of the Internal Quality Control values (ChromSystems, Munich, Germany), was less than 5% for concentrations from 1.8 to 5.2 mg/L. External Quality Assessment was by the Mycophenolate International Proficiency Testing Scheme (Analytical Unit, St George's Hospital Medical School, London, UK).

#### 2.4.6 Statistical analysis

Passing-Bablok analysis was used to compare MPA values, and Bland–Altman analysis was used to evaluate bias and 95% CI limits of agreement. Statistical analyses were performed using MedCalc version 12.3 (MedCalc Software, Ostend, Belgium).

## 3 Results

### 3.1 Validation of the enzymatic assay on the ABX Pentra 400

#### 3.1.1 Functional sensitivity

**Fig. 1:**
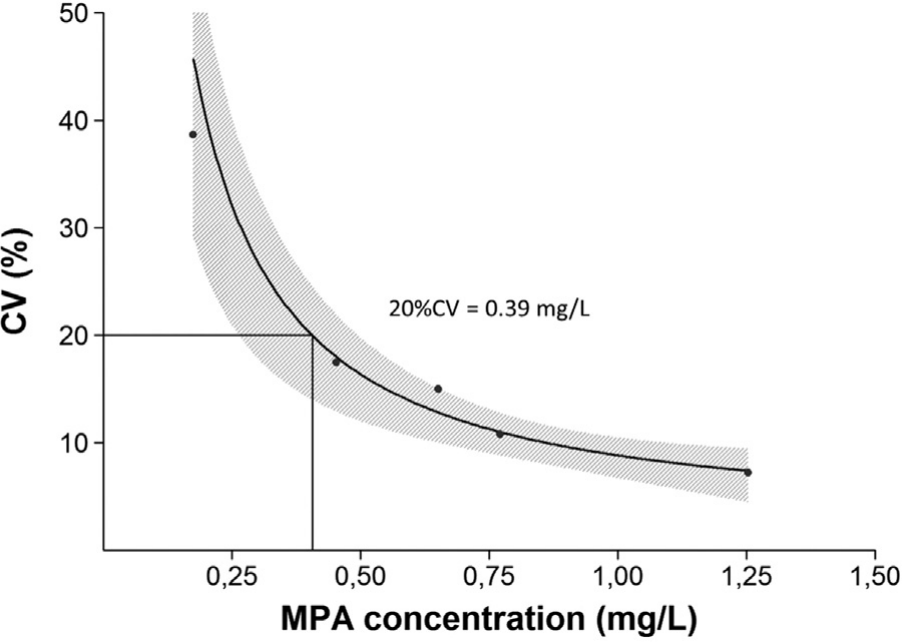
Precision profile and functional sensitivity of the enzymatic assay. The concentration of MPA at which the response in the assay has a total imprecision CV of 20% is the “functional sensitivity”.

The functional sensitivity was determined to be 0.39 mg/L in accordance with results from previously published studies (Brandhorst et al. reported a LLOQ of 0.31 mg/L) [19] (Fig. 1). These results are in good agreement with the LLOQ reported by the manufacturer's package insert (0.40 mg/L).

#### 3.1.2 Precision

Table 1 shows the results of the imprecision studies. For the Siemens EMIT control material, the total CV% were 9.3%, 4.1% and 2.5% for the low control (1.24 mg/L), the medium control (9.3 mg/L), and the high control (14.1 mg/L), respectively. For the Roche control material, the total CV% were 9.7%, 2.5% and 2.9% at <PA concentrations of 0.8, 3.5 and 12.6 mg/L, respectively. In a multicenter evaluation of the enzymatic assay on the COBAS INTEGRA and COBAS C501 analyzers, total imprecision (CV) ranged from 2.8% to 8.7% for the Quality Control 1 (QC1) (0.9 mg/L), from 1.1% to 2.9% for the QC2 (3.5 mg/L), and from 0.9% to 2.1% for the QC3 (12.2 mg/L) [19]. In our imprecision study, the 95% CI of the total CV% overlap these values. In general, a CV of <10% or even <6% is desirable for immunosuppressant drugs methods [25].

**Table 1:** Within-run and total imprecision using the CLSI standard EP5A2.

**Siemens EMIT® MPA controls**			**Within-run**		**Total**	
**IQC levels**	Days	Mean (mg/L)	CV (%)	95% CI	CV (%)	95% CI
**1**	20	1.2	5.5	4.5–6.9	9.3	7.4–11.3
**2**	20	9.3	1.2	1.0–1.6	4.1	2.9–5.3
**3**	20	14.1	1.5	1.2–1.9	2.5	1.8–3.2

**Roche Total MPA controls**			**Within-run**		**Total**	
**IQC levels**	Days	Mean (mg/L)	CV (%)	95% CI	CV (%)	95% CI
**1**	20	0.8	4.3	3.5–5.5	9.7	8.0–12.1
**2**	20	3.5	1.3	1.1–1.6	2.5	2.1–3.1
**3**	20	12.6	1.9	1.6–2.5	2.9	2.4–3.5

#### 3.1.3 Accuracy

The recovery of samples from a proficiency testing scheme is shown in Table 2. Between October 2008 and April 2016, 58 EQA samples were obtained from Analytical Services International. Target/mean values ranged from 0.6 mg/L to 14 mg/L. Mean z-score was 0.2 (Min: −1.7; Max: 1.8) and mean bias was 2.4% (Min: −17.6%; Max:16.7%). The results demonstrated that this adaptation displayed good agreement with the peer group mean.

**Table 2:** Results from EQA samples (Mycophenolate – International Proficiency Testing Scheme, Target Values for “Others”) – M46A/B to M76A/B.

**IQC levels**	n	Mean (mg/L)			Mean bias (mg/L)	Standard deviation of the mean bias (mg/L)
		Participant	Peer grp.			
**<2.5 mg/L**	16	1.84		1.81	0.03	0.16
Nature: target	4	2.25		2.13	0.12	0.1
Nature: pool	12	1.70		1.71	−0.01	0.16
**2.5–10 mg/L**	30	4.91		4.77	0.14	0.25
Nature: target	25	5.25		5.11	0.14	0.26
Nature: pool	5	3.22		3.08	0.14	0.25
**>10 mg/L**	12	12.48		12.10	0.38	0.54
Nature: target	12					

Mean bias and Standard deviation of the mean bias were calculated according to the SH GTA 14, the technical guide for the UM estimation, published by COFRAC (the French accreditation body) in 2011. Ref. [24]

From October 2008 to April 2010, 12 EQA (7 spiked samples) were analyzed simultaneously by then enzymatic and LC-MS assays. Good agreement was obtained between the two assays (Table 3).

**Table 3:** Results from EQA samples (Mycophenolate International Proficiency Testing [PT] Scheme) analyzed simultaneously by the enzymatic and LC-MS assays – M46A/B (October 2008) to M52A/B (April 2010).

PT Samples	Nature	Enzymatic assay (mg/L)	Target (others)	LC–MS assay (mg/L)	Target (HPLC/MS)
M46A	Target	14.0	14	13.0	12.2
M46B	Pool	2.3	2.1	1.9	1.9
M47A	Target	5.4	5.2	4.9	5.0
M47B	Target	5.3	5.1	4.7	5.0
M48A	Target	2.9	2.8	2.7	2.6
M48B	Liver Pool	3.0	2.8	3.0	2.8
M49A	Target	4.3	4.2	No result	4.3
M49B	Target	4.3	4.3	No result	4.3
M50A	Target	13.0	12.7	12.3	12.3
M50B	Pool	2.0	1.9	2.0	1.8
M51A	Pool	1.8	1.8	1.7	1.6
M51B	Target	4.0	4.0	4.0	4.0
M52A	Pool	2.1	1.8	1.7	1.7
M52B	Target	3.2	2.9	2.9	3.0

#### 3.1.4 Uncertainty

The uncertainty of measurement was estimated at 17.3% using the LTUM approach. The quality of this estimate is better as the number of degrees of freedom (number of EQA samples minus two) increases [23]. For immunosuppressive drugs, an uncertainty of measurement (total analytical error) goal of 15% is assumed to be feasible [25].

#### 3.1.5 Carryover effect

Carryover was less than 1% by running a high sample (55 mg/L) followed by a low sample (1.2 mg/L).

### 3.2 Samples from children with idiopathic nephrotic syndrome: comparison with LC-MS method

Three hundred and sixty samples from 42 children with nephrotic syndrome (46 full pharmacokinetic profiles) were analyzed with both techniques. Four samples had MPA concentrations below the enzymatic assay LLOQ (0.4 mg/L) and were removed from the final statistical analysis. The main characteristics and biochemical parameters of the patients are presented in Table 4. Fifteen children received ciclosporin as co-medication.

**Table 4:** Characteristics of children with idiopathic nephrotic syndrome studied.

	Value
Patient characteristics	
Number of patients	42
Sex ration (M/F)	30/12
Age (years)	12 [9–15]
Body weight (kg)	36.4 [27.9–54]
Daily MMF dose (mg/m^2^)	1175 [1134–1259]
CsA co-medication (n=15)	
Number of samples	18
CsA dose (mg/day)	140 [105–195]
CsA trough concentration (µg/L)	89 [67–130]
CsA AUC_0–12 h_ (µg.h/L)	2823 [1391–4163]
Biological values	
Serum protein (g/L)	65 [63–68.8]
Serum albumin (g/L)	40.2 [38–45]
Serum creatinine (µmol/L)	51 [31–111]
Urine protein: creatinine ratio (mg/mmol)	8 ratios>30 mg/mmol

NOTE: Values are expressed as median and interquartile range.

Abbreviations: AUC: area under the curve, CsA, ciclosporin; MMF, mycophenolate mofetil

**Fig. 2:**
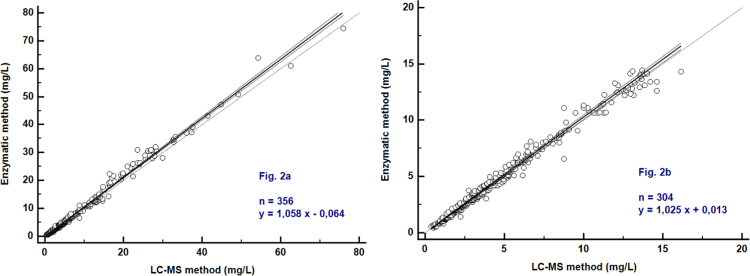
Passing-Bablok regression analysis of the enzymatic method compared to LC-MS. Comparison using plasma samples with MPA concentrations >0.4 mg/L (Fig. 2a) or with MPA concentrations within the linearity range of the enzymatic method (0.4–15 mg/L) (Fig. 2b). The full line is the regression-line. The two dashed lines show the 95% confidence interval and the point line is slope=1.

As shown in Fig. 2a, the two methods correlated strongly (Spearman's rho=0.996) with a Passing-Bablok correlation function: [Enzymatic assay]=1.058 [LC-MS] – 0.064 (n=356). The 95% CI of the slope was 1.044–1.074. The Bland-Altman plot analysis demonstrated that the MPA enzymatic method tended to slightly overestimate the MPA value (the average percent bias was 4.1%) (Fig. 2b).

**Fig. 3:**
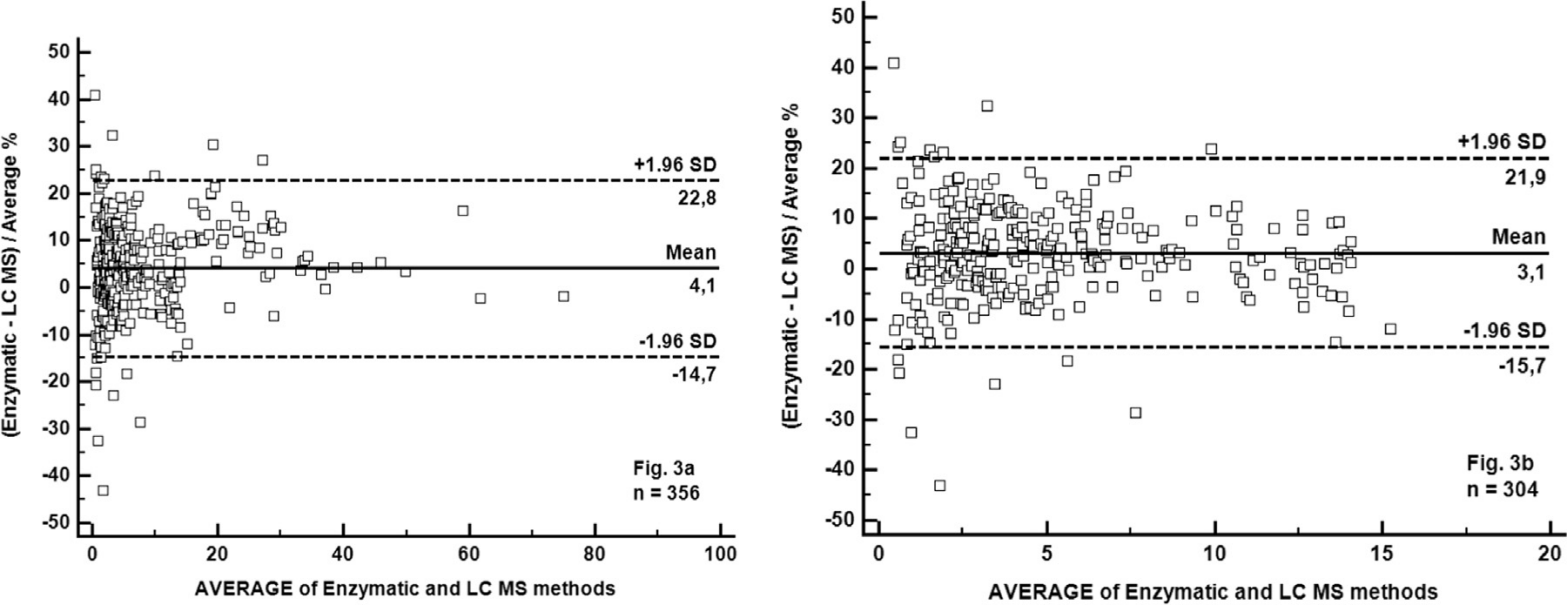
Bland-Altman plot of the enzymatic method compared to LC-MS. Comparison using plasma samples with MPA concentrations >0.4 mg/L (Fig. 3a) or with MPA concentrations within the linearity range of the enzymatic method (0.4–15 mg/L) (Fig. 3b). The two dashed lines are the 95% confidence interval of mean of differences.

Limiting comparison to the 304 samples with MPA concentration values within the linearity range of the enzymatic assay (0.4–15 mg/L), the Passing-Bablok correlation function was: [Enzymatic assay]=1.025 [LC–MS]+0.013. The average percent bias was 3.1% (Fig. 3a and b).

Median AUC_0–12h_ of MPA calculated by the two methods and dose-normalized to 1200 mg/m^2^/day were 59.6 mg h/L (Interquartile Range [IQR]: 47.3–71.9) and 56.0 mg h/L (IQR: 45.6–71.4), respectively.

These data were also stratified for (i) the presence or (ii) absence of ciclosporin co-medication:

(i) [Enzymatic assay]=1.082 [LC–MS] −0.084; n=143
(ii) [Enzymatic assay]=1.046 [LC–MS] −0.043; n=213

The 95% CI of the slopes overlap: (i) 1.057–1.105 and (ii) 1.030–1.067.

Note regression analysis of the enzymatic method vs. LC with diode array detection is provided as a Supplementary file.

## 4 Discussion

Several commercial immunoassays have been developed for the determination of the plasma MPA using EMIT (Enzyme multiplied immunoassay technique), PETINIA (Particle Enhanced Turbidimetric Inhibition Immunoassay) or CEDIA (cloned enzyme donor immunoassay) -based procedures. Immunoassays are particularly convenient because they can be run on various automated clinical chemistry analyzers found in many laboratories worldwide. However, a well-described problem is the clinically relevant overestimation of the MPA concentrations by EMIT [26], PETINIA [27] or CEDIA [14] immunoassays (e.g. the CEDIA immunoassay overestimates MPA concentrations on average by 36%) [14]. This could partly be explained by cross-reactivity with AcMPAG [28]. Because cross-reactivity is concentration-dependent and AcMPAG concentrations vary within and between patients, a simple conversion of MPA concentrations determined by immunoassays to concentrations measured by chromatographic methods is not possible.

So far, therapeutic ranges for MPA therapy are mainly based on specific chromatographic techniques. In clinical laboratories, High-Performance Liquid Chromatography (HPLC), especially in combination with tandem mass spectrometry (LC-MS/MS), has become the standard procedure to quantify MPA. In 2013 under the auspices of the IATDMCT, a web-based survey was conducted to systematically document current practices for the TDM of immunosuppressant drugs in clinical laboratories. For TDM of mycophenolic acid, 26% of the laboratories used immunoassays, 37% used LC-MS/MS and 37% used HPLC/UV (n=52) [25].

The Roche Total Mycophenolic Acid® assay was developed as an alternative to chromatographic methods. A multicenter evaluation demonstrated a highly reliable assay that showed good agreement with HPLC-UV and LC-MS/MS methods [19]. At least four others studies demonstrated the reliability of this enzymatic assay in different populations of allograft recipients given MMF [18], [29], [30], [31].

The only official protocols for the enzymatic assay are on the COBAS INTEGRA (Roche Diagnostics Systems) analyzers. Here we have developed and validated an application protocol for the ABX Pentra 400 analyzer. The ABX Pentra 400 analyzer offers open channels for customer-specific applications and it is devoted to automated toxicology and TDM drug assays in our laboratory.

The functional sensitivity (0.39 mg/L) is adequate for clinical application considering the therapeutic range for pre-dose MPA concentration in renal transplant patients during the initial phase in association with CsA: 1–3.5 mg/L [19]. The analyte quantification should be at least one-third to half of the lower limit of the target concentration window [25]. However, a LLOQ of 0.2 mg/L should be targeted for MPA [25].

The within-run and total imprecision of the enzymatic assay were within the design targets set for the TDM of MPA [25]. In general, for immunosuppressive drugs methods, a total imprecision CV of ≤10% should be aimed for [25]. However, in our precision study performed according to CLSI document EP5-A2, the total imprecision was 9.7% for the Roche Total MPA control Level 1 (0.8 mg/L; human serum matrix) and 9.3% for the Siemens EMIT MPA control Level 1 (1.2 mg/L; aqueous/bovine serum albumin matrix), slightly above the target total imprecision reported for this level of concentration [19]. The recovery of samples (n=58) from the International Proficiency Testing Scheme was in accordance with the acceptance criteria for enzymatic and chromatographic assays. The enzymatic method on the ABX Pentra 400 analyzer brings several advantages to the routine lab, limiting the time needed for every determination and reducing the need of dedicated personnel to perform the assay. The added convenience of interfacing to the LIS removed the manual transcription process for both quality control and patient results. This assay has the disadvantage that the determination of non-trough concentrations, and especially the analysis of 1–2 h post-dose samples, requires frequent use of manual dilutions (about 15% of samples). Automatic dilution of samples with an “open-channel” diluent was unfortunately not possible on the ABX Pentra 400 analyzer.

The in-use reagent stability (stability of reagents after being unpacked and putted into the Pentra 400 reagent rack) was only indirectly verified in the laboratory through quality control behaviour. So, the in-use manufacturer reagent stability claims (after being first opened) cannot be guaranteed. The high cost of reagents is another disadvantage.

The recently published study of Hackl et al. [10] suggests that a plasma MPA-AUC_0–12h_ lower than 44.6 mg h/L was a risk factor for future relapses in children with idiopathic nephrotic syndrome (NS) treated with MMF (91% sensitivity, 57% specificity, p=0.02). The results of this study was in accordance with previously published studies showing an efficacy of MPA proportional to the drug exposure in pediatric NS [9], [12]. Based on these findings and given the substantial inter- and intra-patient variability of MPA exposure, it would appear that MMF therapy should be subject to TDM to avoid over- and under-dosing. However, the enzymatic assay has not been evaluated in children with NS. To show the interchangeability of enzymatic assay and LC-MS in children with NS treated with MMF, 356 samples were analyzed with both techniques. The enzymatic assay showed an overall excellent agreement to the LC-MS reference method. However, it tends to slightly overestimate MPA concentrations (median overestimation of 4.1%) as previously reported in other studies in transplant patients [18], [29], [30], [31]. This overestimation was not considered to be clinically relevant.

## 5 Conclusion

The Roche Total Mycophenolic Acid® assay on the ABX Pentra 400 analyzer offers the user many benefits provided by an automated assay, e.g., rapid sample throughput, minimum operator intervention, ready-to-use liquid reagents, LIS interface. However, the non-availability of an automatic post sample dilution with an “open-channel” diluent is problematic. The enzymatic assay showed an excellent correlation with LC–MS in children with INS treated with MMF. The bias (4.1%) was similar to the bias observed in different populations of allograft recipients given MMF.
