# Supplementary material for: The Roche Total Mycophenolic Acid® assay: An application protocol for the ABX Pentra 400 analyzer and comparison with LC–MS in children with idiopathic nephrotic syndrome
Source: Pract Lab Med. 2017 Jan 4;7:19–26. doi: 10.1016/j.plabm.2016.12.002 (PMC5575364; doi:10.1016/j.plabm.2016.12.002)
Supplement: Supplementary file 1 — Supplementary material [file mmc1.docx]

| 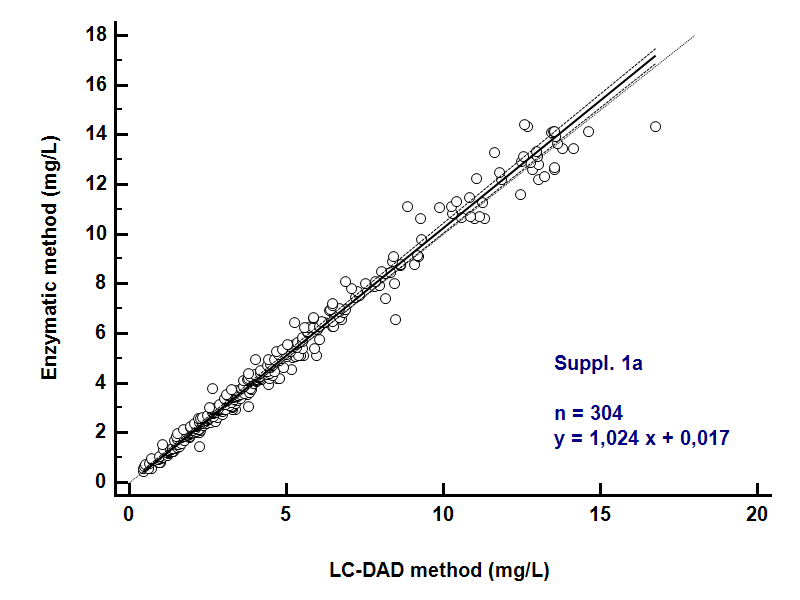 | 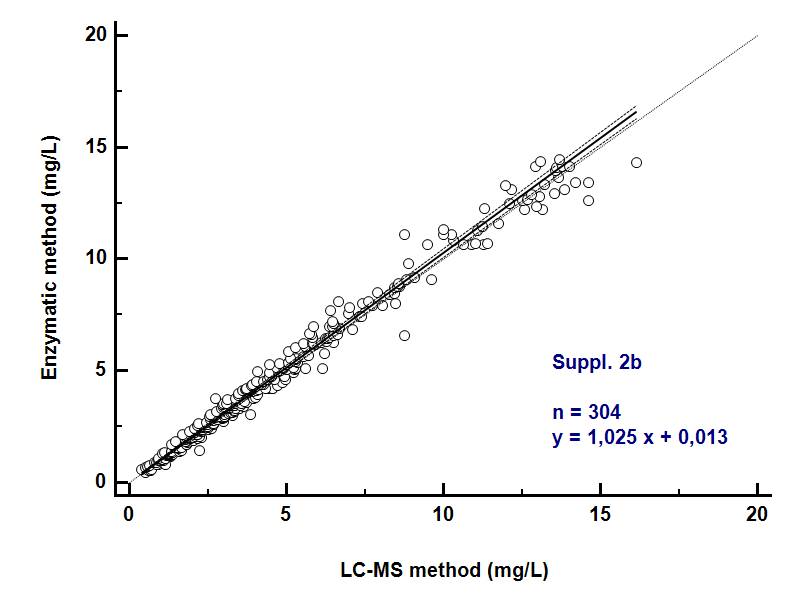 |  |  |
| --- | --- | --- | --- |
|  | |  |  |

**Supplement 1** Passing-Bablok regression analysis of the enzymatic method compared to LC method with DAD detection (252 nm) (Suppl. 1a) or MS spectrometry (Suppl. 1b). The MPA concentrations were within the linearity range of the enzymatic method (0.4-15 mg/L). The full line is the regression-line. The two dashed lines show the 95% confidence interval and the point line is slope = 1.
